# Feasibility and safety of early discharge after transfemoral transcatheter aortic valve implantation – rationale and design of the FAST-TAVI registry

**DOI:** 10.1186/s12872-017-0693-0

**Published:** 2017-10-10

**Authors:** Marco Barbanti, Jan Baan, Mark S. Spence, Fortunato Iacovelli, Gian Luca Martinelli, Francesco Saia, Alessandro Santo Bortone, Frank van der Kley, Douglas F. Muir, Cameron G. Densem, Marije Vis, Martijn S. van Mourik, Lenka Seilerova, Claudia M. Lüske, Peter Bramlage, Corrado Tamburino

**Affiliations:** 1 Catania Division of Cardiology, Ferrarotto Hospital, University of Catania, Via Salvatore Citelli 6, Catania, Italy; 2 Department of Cardiology, Academic Medical Center, Amsterdam, The Netherlands; 3 Cardiology Department, Royal Victoria Hospital, Belfast, UK; 4 Interventional Cardiology Service, “Montevergine” Clinic, Mercogliano, Italy; 5 Division of Cardiology, Department of Advanced Biomedical Sciences, University of Naples “Federico II”, Naples, Italy; 6 Novara Department of Cardiac Surgery, Clinica San Gaudenzio, Novara, Italy; 7 Cardiovascular and Thoracic Department, S. Orsola-Malpighi University Hospital, Bologna, Italy; 8 Department of Interventional Cardiology, University of Bari “Aldo Moro”, Bari, Italy; 9 Department of Cardiology, Leiden University Medical Center, Leiden, The Netherlands; 10 Cardiothoracic Division, The James Cook University Hospital, Middlesbrough, UK; 11 Department of Interventional Cardiology, Papworth Hospital, Cambridge, UK; 12 Edwards Lifesciences, Prague, Czech Republic; 13 Institute for Pharmacology and Preventive Medicine, Cloppenburg, Germany

**Keywords:** Aortic stenosis, Hospitalisation, Length of stay, Cost-effectiveness, Transfemoral, SAPIEN

## Abstract

**Background:**

There is an increasing trend towards shorter hospital stays after transcatheter aortic valve implantation (TAVI), in particular for patients undergoing the procedure via transfemoral (TF) access. Preliminary data suggest that there exists a population of patients that can be discharged safely very early after TF-TAVI. However, current evidence is limited to few retrospective studies, encompassing relatively small sample sizes.

**Methods:**

The Feasibility And Safety of early discharge after Transfemoral TAVI (FAST-TAVI) registry is a prospective observational registry that will be conducted at 10 sites across Italy, the Netherlands and the UK. Patients will be included if they have been scheduled to undergo TF-TAVI with the balloon-expandable SAPIEN 3 transcatheter heart valve (THV; Edwards Lifesciences, Irvine, CA). The primary endpoint is a composite of all-cause mortality, vascular-access-related complications, permanent pacemaker implantation, stroke, re-hospitalisation due to cardiac reasons, kidney failure and major bleeding, occurring during the first 30 days after hospital discharge. Patients will be stratified according to whether they were high or low risk for early discharge (≤3 days) (following pre-specified criteria), and according to whether or not they were discharged early. Secondary endpoints will include time-to-event (Kaplan–Meier) analysis for the primary outcome and its individual components, analysis of the relative costs of early and late discharge, and changes in short- and long-term quality of life. Multivariate logistic regression will be used to identify factors that indicate that a patient may be suitable for early discharge.

**Discussion:**

The data gathered in the FAST-TAVI registry should help to clarify the safety of early discharge after TF-TAVI and to identify patient and procedural characteristics that make early discharge from hospital a safe and cost-effective strategy.

**Trial registration:**

ClinicalTrials.gov Identifier: NCT02404467 (registration first received March 23rd 2015).

## Background

Transcatheter aortic valve implantation (TAVI) is a feasible alternative to surgical aortic valve replacement (SAVR) for patients who are at prohibitively high risk for open surgery. More recently, there has been a trend towards performing TAVI in lower-risk patients, with similar rates of all-cause mortality reported in a randomised trial comparing TAVI with SAVR in intermediate risk patients [1]. The cost-effectiveness of TAVI compared to SAVR has been shown to be acceptable in high-risk patients, especially when transfemoral (TF) access is utilised [2]. However, in a real-world setting, the high cost of TAVI, mainly due to the price of the transcatheter heart valve (THV), limits extension of the procedure to lower risk patients [3].

Hospitalisation is the main contributor to the costs of SAVR, and the second largest contributor to the costs of TAVI [4]. While there is limited scope for reducing the length of hospital stay after open surgery, increasing use of a minimalist approach to TAVI has the potential to significantly reduce the time to discharge. When TF access is used, the procedure can often be performed in a catheterisation laboratory, with use of local anaesthesia and conscious sedation rather than general anaesthesia. This approach reduces the costs of the procedure itself and allows for a shorter stay in the intensive care unit (ICU) and the potential for early discharge from hospital [5–7]. Indeed, there has been a significant decreasing trend in length of hospital stay after TAVI [8, 9], with a recent study reporting an average of 4 days after TF-TAVI at a centre that actively pursued early discharge [10].

A number of studies have evaluated the safety of early discharge after TAVI [8, 11, 12]. Lauck et al. retrospectively evaluated data from 393 TAVI patients, 38% of whom had been discharged within 48 h after their procedure. They found no differences in terms of 30-day mortality, rehospitalisation or disabling stroke between the early and standard discharge groups [11]. Similarly, Durand et al. reported discharge within 72 h for 36% of their 337 TF-TAVI patients, with no difference in 30-day mortality or rehospitalisation [8].

In a small prospective study (*N* = 130), early discharge was specifically targeted after elective TF-TAVI [10]. A total of 59% of patients were successfully discharged within 72 h, with one death and 3 cases of rehospitalisation occurring during the subsequent 30 days. While no death occurred in the patients that were discharged after 72 h, 7 required rehospitalisation. In a cohort of 120 patients that underwent TAVI at a single centre, 21.7% of patients were discharged on either the same day as the procedure or the following day, with a further 32.5% discharged at 2 or 3 days [6]. There were no deaths within 30 days for any of these patients, while mortality was 5.5% for those that were discharged after 4 days. Rehospitalisation rates did not differ significantly between groups.

These studies have provided preliminary data in support of the feasibility and safety of early discharge after TF-TAVI. However, if this approach is to be more widely adopted, there is a clear need for larger, prospective multicentre studies. A substantial cohort of TAVI patients would also allow for evaluation of baseline and procedural characteristics that may indicate suitability for early discharge, further decreasing the associated risks. Furthermore, the rapid advancements being made in THV and implantation technologies make up-to-date information essential.

The Feasibility And Safety of early discharge after Transfemoral-TAVI (FAST-TAVI) registry has been designed in order to provide contemporary data regarding early discharge after TAVI. This prospective, multicentre study will evaluate patients undergoing TF-TAVI with the latest generation of the balloon-expandable SAPIEN THV (SAPIEN 3; Edwards Lifesciences). In addition to assessing adverse outcomes after discharge, the dataset will enable the identification of criteria that will allow safe early discharge of patients after TF TAVI.

## Methods/design

FAST-TAVI is an observational, prospective, multicentre registry that will be performed at 5 sites in Italy (Catania, Bari, Novara, Bologna and Mercogliano), 2 sites in the Netherlands (Amsterdam & Leiden), and 3 sites in the UK (Belfast, Cambridge, Middlesbrough). Approximately 50 patients undergoing TF-TAVI with the SAPIEN 3 THV will be enrolled at each site.

### Patients

Patients undergoing TF-TAVI with the SAPIEN 3 THV (Edwards Lifesciences) will be enrolled on a consecutive basis. The decision to perform this procedure will be made by the Heart Team at each institution according to standard practice; it will not be influenced in any way by the investigators. Beyond the applicable criteria of the device Instructions for Use, no other inclusion or exclusion criteria will be applied.

### Data collection

Data will be collected prospectively according to the timetable set out in Table 1, and will be entered in a standardised case report form (CRF). At baseline, demographic and clinical characteristics will be documented. Laboratory data from blood and urine analysis will be collected and an echocardiogram and an ECG will be performed. Patients will also undergo a full physical examination. A mini-mental state examination (MMSE) will be carried out and patients will be asked to complete the SF-12 QoL (Quality of life) questionnaire (version 2.0). Procedural characteristics, including any complications, will be collected. Post-procedure, patients will be monitored according to standard practice. An echocardiogram and an ECG will be acquired within 2 h of the procedure, and at least once prior to discharge. A physical examination and blood and urine analysis will be performed at daily intervals until discharge.

**Table 1:** Data collection timetable

	Baseline	Procedure (up to 2 h post-TAVI)	Day 1	Day 2^c^	Day 3^c^	Discharge	30 ± 12 days	12 months
Informed consent	x							
Demographics	x							
Clinical characteristics	x							
Physical examination^a^	x		x	x	x	x	x	x
Laboratory analysis^b^	x		x	x	x	x		
Current medication	x					x	x	x
ECG	x	x	x	x	x	x	x	x
Echocardiogram	x	x	x			x	x	x
MMSE	x							
SF-12	x						x	x
Clinical event assessment		x				x	x	x

Legend: ECG, electrocardiogram; MMSE, mini-mental state examination; SF-12, short-form-12 quality of life questionnaire. ^a^Includes symptoms, mobility, self-care; ^b^includes blood and urine analysis (complete blood count, electrolytes, renal function etc.); ^c^if still in hospital

Patients will be discharged when it is deemed appropriate by the treating physician. This will be unaffected by their participation in the registry. The length of hospital stay will be documented. Follow-up visits will be conducted in accordance with hospital protocol. Data regarding events during the first 30 ± 12 days after discharge will be collected at next visit after this time point. These will include the components set out in the Valve Academic Research Consortium (VARC)-2 consensus document [13]. An echocardiogram and an ECG will be obtained and a full physical examination will be carried out. Furthermore, patients will be asked to again complete the SF-12 QoL questionnaire.

Further follow-up information will be collected at 12 months post-TAVI. This will include the results of a physical examination, blood and urine analysis, echocardiography and an ECG. Any adverse events or rehospitalisation during the 12 months since TAVI will be recorded.

### Patient stratification

Patients will be stratified when data collection for all patients is complete. A patient will be classified as being at low risk for early discharge if they fulfil all of the criteria at the point of leaving hospital, as displayed in Table 2. The patients will be further stratified according to whether they were discharged early (≤3 days post-TAVI) or late (<3 days). This will give 4 groups for comparison purposes. Further time points (e.g. stratification at hospital admission) as well as cut-offs (1, 2 or 4 days etc.) will be explored once data are available.

**Table 2:** Patient stratification

A patient will be classified as being at low risk for early discharge if they fulfil all of the following criteria at the point of leaving hospital:
New York Heart Association (NYHA) class ≤ II
No chest pain attributable to cardiac ischaemia
No untreated major arrhythmias
Complications on day 0 to 1, but free of signs or symptoms on day 3
No fever during the last 24 h (infection-related)
Independent mobilisation and capability of self-care
Preserved diuresis (>40 ml/h during the last 24 h)
No unresolved acute kidney injury type 3 (according to VARC-2 criteria)
No red blood cell transfusion during the last 72 h
Stable haemoglobin in 2 consecutive samples (defined as a decrease of no more than 2 mg/dl)
No stroke or transient ischaemic attack (TIA)
No sign of systemic inflammation or infection (clinic or laboratory)
No haemodynamic instability

### Primary endpoint

The primary endpoint is a composite of all-cause mortality, vascular-access-related complications, permanent pacemaker implantation, stroke, re-hospitalisation due to cardiac reasons, kidney failure and major bleeding, occurring during the first 30 days after hospital discharge.

Cumulative and time-dependent (Kaplan–Meier) incidence of the primary endpoint will be compared between the 4 groups stratified according to suitability for early discharge (according to protocol) and actual discharge.

### Secondary endpoints

The incidence of the individual components of the primary outcome (between discharge and 30 days) will be evaluated for the 4 groups. Time-dependent (Kaplan–Meier) incidence of the primary outcome and its individual components will also be assessed between discharge and 12 months after TAVI.

Multivariate analysis will be performed in order to identify procedural outcomes associated with incidence of the primary endpoint in the patients that were discharged early. A further analysis will be performed to identify factors predictive of early discharge.

Other endpoints will include the length of ICU and overall hospital stay; the QoL scores at baseline, 30 days and 12 months. Other exploratory endpoints may be investigated.

### Statistics

As there are few reliable data on early discharge after TAVI, no formal sample size calculation was performed. Based on rates of TAVI procedures being performed, it was estimated that approximately 50 patients could be recruited in one year at each site.

Intent-to-treat analysis, defined as all patients enrolled in the registry, will be employed. Subjects will be considered registry participants when they enter the catheterisation laboratory/hybrid suite/operating room. Descriptive data summaries will be used to present and summarise the collected data. For categorical variables, frequency distributions will be given. For numeric variables, means and standard deviations or medians and interquartile ranges will be calculated, depending on data distribution. Kaplan–Meier analysis will be performed for time-to-event outcomes. Multivariate logistic regression will be performed to identify predictors of the primary endpoint in the patients discharged early, and predictors of early discharge. Variables entered into the analysis will include baseline characteristics and periprocedural complications.

## Discussion

The FAST-TAVI registry has been designed to provide a registry of prospectively collected data that can be used to elucidate the benefits and risks of early discharge after TF-TAVI. Analysis of the results should enable identification of certain patient and procedural characteristics that indicate whether a patient requires further in-hospital monitoring or whether they could safely be discharged within just a few days after the TAVI procedure.

Preliminary data from previous studies suggest that there exists a population of patients that can be safely discharged soon after undergoing uncomplicated TAVI via the TF route [6, 8, 10, 11]. However, the human and financial costs associated with inappropriate early discharge could be immense. Complications after TAVI include bleeding, stroke and kidney injury, each associated with a mortality risk. Furthermore, all patients that undergo TAVI are at high risk for death during open cardiac surgery. They are generally elderly and display multiple comorbidities and frailty, providing an even greater risk of mortality. In addition, unplanned rehospitalisation after TAVI is expensive and so may counteract the cost savings made by discharging a patient early [14]. Studies evaluating readmission after TAVI have consistently found that heart failure is the most common cause, although the relative contributions of other factors varied [14, 15]. Furthermore, high proportions of patients were hospitalised for non-cardiovascular reasons, highlighting the complex nature of this elderly and comorbid population.

In an attempt to identify factors that indicate that an individual patient is suitable for early discharge after TAVI, Durand et al. reviewed the records of all patients that underwent TF-TAVI using the SAPIEN XT THV during a 4-year period [8]. Of the baseline and procedural characteristics that were entered into their multivariate analysis, a requirement for blood transfusion(s) and previous balloon aortic valvuloplasty were predictive of late discharge, while a pre-existing pacemaker was associated with early discharge. There was also a wide variety of univariate predictors that may have proved more influential in a larger population. The FAST-TAVI registry will build on these initial data while investigating the most recent of the SAPIEN THVs, the SAPIEN 3.

Often overlooked aspects of recovery after TAVI are patient comfort and the mental and emotional aspects that affect their QoL during the first few days and weeks. In a recent study looking into self-reported health and QoL changes during the first month after TAVI, Olsen et al. reported a significant improvement in the physical component summary of the SF-12 questionnaire, but not in the mental component summary [16]. The main contributing factors to the insignificant increase in the mental component were social and emotional, which is in agreement with a previous study by Krane et al. [17]. Reynolds et al. reported a significant improvement in both physical and mental scores at 6 months and one-year post-TAVI compared to baseline; however, the mental component only increased slightly during the first 30-days of follow-up [18]. It is possible that early discharge from hospital may help to improve patients’ emotional wellbeing in the first month after TAVI. In order to evaluate this hypothesis, the SF-12 questionnaire will be completed at baseline, 30 days and 12 months in the FAST-TAVI registry.

### Potential limitations

While the multinational nature of this registry increases the applicability of the findings to other countries, the differences in healthcare systems may also introduce some difficulties. This is of particular significance when evaluating the financial implications of early discharge. Furthermore, standard procedural and aftercare protocols are likely to vary between countries, and possibly between institutions within a country. However, one significant advantage of the present registry is that all patients will receive the same THV (SAPIEN 3) via the same access route (TF), reducing the variability common to the majority of prior TAVI studies.

### Potential clinical impact

The knowledge acquired from the FAST-TAVI registry should help to elucidate the relative risks and benefits of discharging a patient early after TF-TAVI. This not only includes the clinical implications for the patient, but also takes into account their QoL. Furthermore, with hospitalisation contributing significantly to the overall cost of a TAVI procedure, the potential for extending its cost-effectiveness to lower-risk patients can be explored.

## Abbreviations

CRF: Case report form
FAST-TAVI: The Feasibility and Safety of early discharge after Transfemoral TAVI
ICU: Intensive care unit
MMSE: Mini-mental state examination
QoL: Quality of life
SAVR: Surgical aortic valve replacement
SF-12: Short-form-12 quality of life questionnaire
TAVI: Transcatheter aortic valve implantation
TF: Transfemoral
THV: Transcatheter heart valve
VARC-2: Valve Academic Research Consortium-2
